# Oat Yogurts Enriched with Synbiotic Microcapsules: Physicochemical, Microbiological, Textural and Rheological Properties during Storage

**DOI:** 10.3390/foods11070940

**Published:** 2022-03-24

**Authors:** Liliana Luca, Mircea Oroian

**Affiliations:** Faculty of Food Engineering, Stefan cel Mare University of Suceava, 720229 Suceava, Romania; liliana.luca@usm.ro

**Keywords:** *Lactobacillus*, oligofructose, probiotic, prebiotic, synbiotic, starch

## Abstract

The aim of this study was to evaluate the influence of synbiotic microcapsules on oat yogurt’s properties. For this study, four different microcapsules were added into the oat yogurt and the modifications were studied for 28 days. Microbiological analysis was used to analyze the effect of different factors on the microencapsulated probiotic population in the product. Those factors are: the technological process of obtaining microcapsules; the type of prebiotic chicory inulin (INU), oligofructose (OLI) and soluble potato starch (STH); the prebiotic concentrations in the encapsulation matrix; the technological process of obtaining yogurt; and the yogurt storage period, gastric juice action and intestinal juice action. The experimental data show that oat yogurt containing synbiotic microcapsules has similar properties to yogurt without microcapsules, which illustrates that the addition of synbiotic microcapsules does not change the quality, texture or rheological parameters of the product. Oat yogurt with the addition of synbiotic microcapsules can be promoted as a functional food product, which, in addition to other beneficial components (bioactive compounds), has in its composition four essential amino acids (glycine, valine, leucine and glutamine acids) and eight non-essential amino acids (alanine, serine, proline, asparagine, thioproline, aspartic acid, glutamic acid and α-aminopimelic acid). After 28 days of storage in refrigerated conditions, the cell viability of the microcapsules after the action of the simulated intestinal juice were: 9.26 ± 0.01 log10 cfu/g, I STH (oat yogurt with synbiotic microcapsules—soluble potato starch); 9.33 ± 0.01 log10 cfu/g, I INU, 9.18 ± 0.01 log10 cfu/g, I OIL and 8.26 ± 0.04 log10 cfu/g, IG (oat yogurt with microcapsules with glucose). The new functional food product provides consumers with an optimal number of probiotic cells which have a beneficial effect on intestinal health.

## 1. Introduction

Over the years, the probiotics market has focused on dairy products (yogurt and other fermented products), which are the predominant commercial food matrices of probiotic cultures. However, people who adopt a vegetarian lifestyle and people with health problems (allergy to milk proteins, lactose intolerance and avoiding saturated fatty acids and cholesterol in high concentrations) have changed the interest of the scientific community [1]. Therefore, there has been an increase in the amount of research into new natural components and the development of new non-dairy products that has led to finding innovative recipes in the food industry [2]. Thus, plant matrices have been suggested as potential probiotic “vectors” [3]. Fruit juices and teas or fermented beverages [4] are favorable matrices for the development of these cultures. Drinks from various vegetables (soy) and cereals (wheat, barley, corn, rice, oats, quinoa, millet, rye, sorghum and chia) were made in order to replace traditional dairy drinks [5]. Thus, cereals are rich sources of energy, minerals and vitamins and are being grown and consumed worldwide. It is important to mention that prebiotics are present in cereals [6] and they help maintain healthy intestinal conditions and can stimulate the growth of probiotics and other beneficial intestinal microorganisms (*Bifidobacterium* spp., *Lactobacillus* spp., etc.). Therefore, products that have added value through the content of probiotic cultures are distinguished by their use due to their beneficial effect on human health. The effectiveness of probiotics is associated with their cell viability in food, which depends on the presence of several factors in the production process, storage, etc. To improve and support cell viability, several approaches have been performed, such as the selection of probiotic strains and compatible food matrices and developing synbiotic combinations [7] by adding prebiotics to support viability and stimulate probiotic growth. Oats can be chosen as a matrix for probiotic cultures due to their nutritional value and health benefits. Compared to other types of cereals, oats contain more soluble fiber, which provides an extended feeling of satiety and leads to a slower digestion. The soluble fiber in whole oats contains a class of polysaccharides known as β-D-glucan. β-D-glucans contain a class of indigestible polysaccharides that are widely found in nature (cereals, yeast, bacteria, algae and fungi). Unlike other types of β-glucans, oats are a soluble fiber. Oat β-glucan is composed of mixed-link polysaccharides. This means that the bonds between D-glucose or D-glucopyranosyl units are β-(1,3) or β-(1,4) [8,9,10,11,12].

The literature presents numerous nutritional benefits of oats, but there are very few studies on the use of oats as a dietary matrix for probiotics. Thus, Gupta and Bajaj [13] developed a fermented product based on oatmeal, Mårtensson et al. [14] obtained an oat-based fermented beverage from a product called ADAVENA M40, Bernat et al. [15] made an oat milk product fermented with *Lactobacillus reuteri ATCC 55,730* and *Streptococcus thermophilus CECT 986*, Gupta et al. [8] developed a functional oatmeal drink, and Mantzouridou et al. [16] made an emulsion for the preparation of a low-oil salad based on oatmeal to support the probiotic growth of *Lactobacillus paracasei* subsp. *paracasei DC412*. 

Given the need for functional non-dairy foods, the present study was performed to obtain an oat yogurt enriched with probiotic microcapsules with glucose and synbiotic microcapsules—microcapsules with chicory inulin, microcapsules with oligofructose and microcapsules with potato starch. This type of synbiotic product, oat yogurt, would be a novelty in the food industry, because probiotics provide benefits to the health of the consumer if they are in sufficient numbers. The prebiotics are used for a microcapsules matrix that protects and nourishes the probiotics inside it during the technological process, storage period and also during the action of the digestive juices. In several studies, such as those described by Gueimonde et al. [17], Ibrahim et al. [18] and Lin et al. [19], it is reported that there are commercial products, including fermented dairy products, yogurts, granular powders, etc., which contain less than the cell concentration written on the label [20]. The aim of the study was to ensure that the synbiotic products have functional benefits for the consumer, have similar properties with yogurts and also that they are accepted by consumers.

## 2. Materials and Methods

### 2.1. Starter Cultures

A thermophilic culture distributed by Chr. Hansen (Hørsholm, Denmark) FD-DVS YC-350 YoFlex^®^ that contains *Lactobacillus delbrueckii* subsp. *bulgaricus* and *Streptococcus thermophilus* was used.

### 2.2. Vegetable Oat Beverage

The oat beverage was obtained from whole oat grains Solaris Plant S.R.L., Bucharest, Romania, iodized salt 0.2%, sugar 4.5%, virgin coconut oil 1.5% (Solaris Plant S.R.L., Bucharest, Romania) and xanthan gum 0.01%. The characteristics of oat beverage per 100 mL are: 2.2% lipids; 0.2% salt; 11.6% carbohydrates; 1.4% fiber; 0.9% protein.

### 2.3. Synbiotic Microcapsules

Four types of synbiotic microcapsules (Table 1) studied and described by Luca and Oroian [21] were used, which have, in their matrix, a concentration of 2% sodium alginate, 2% prebiotic and a mixed cultures of *Lactobacillus (L. casei*, *L. rhamnosus* and *L. plantarum*). 

The prebiotics was represented by: soluble potato starch (STH) from Sigma-Aldrich (Germany), and chicory inulin (INU)/Fibruline^®^DS2—98%/inulin and oligofructose (OLI) from Cosucra (France). To obtain the emulsion used to form synbiotic microcapsules, the following materials were used: sodium alginate 2%, sunflower vegetable oil 10%, prebiotic 2%, and sterile water up to 100 mL. All these % are in *w*/*v*. These materials were mixed with a homogenizer (OV5 Homogenizer, Velp Scientifica SRL, Usmate Velate, Italy) at 10,000 U/min for 15 min. Ten milliliters of pellet (10^14^ cfu/mL) was added to the emulsion and mixed on a magnetic plate at 150 rpm for 15 min. The microcapsules have been obtained through the extrusion method using a peristaltic pump (Masterflex 77200-62Easy-Load Pump Head) and needles with a diameter of 0.29 mm were used according to the protocol described by Luca and Oroian [22].

### 2.4. Reagents

Reagents: BioXtra sodium chloride ≥ 99.5%, BioXtra potassium chloride ≥ 99%, bile salts, baking soda NaHCO_3_ from Sigma-Aldrich (Schnelldorf, Germany), calcium chloride CaCl2 × 6H_2_O (Lach—Ner Company, Neratovice, Czech Republic) and pepsin (Across Organics, Madrid, Spain), as well as MRS agar culture medium (VWR International Chemicals, Leuven, Belgium) purchased from East Corp (Iasi, Romania).

### 2.5. Preparation of Synbiotic Yoghurt

Prior to the preparation of vegetable yogurt, the oat beverage was obtained. The oat beverage was prepared from whole oat grains and water using the Biovita VEGALUX device (Cluj Napoca, Romania) for vegetable beverages. After obtaining the oat beverage, the temperature was maintained between 85 and 90 °C for 15 min. At this point, the rest of the ingredients were added, so that in the end, a 16% dry matter content was the result. After the oat beverage’s temperature reached 43 °C, the starter culture FD-DVS YC-350 YoFlex^®^ Chr. Hansen (Hørsholm, Denmark) was inoculated according to the manufacturer’s instructions. Separately, 1 g synbiotic microcapsules (12 log10 cfu/100 mL jar) were placed into the jars, and the oat beverage with the starter culture was added after. The jars were thermostated at 45 °C for 16 h in the thermostat (MEMMERT GmbH + Co. KG, Schwabach, Germany). At the end of the thermostating period, the yogurt samples were cooled to room temperature (25 ± 2 °C) and then refrigerated (4 °C) for 12 h. The analyses were performed after 12 h of refrigeration. 

### 2.6. Survivability of Microencapsulated Probiotics in Vegetable Yogurt

**Figure 1:**
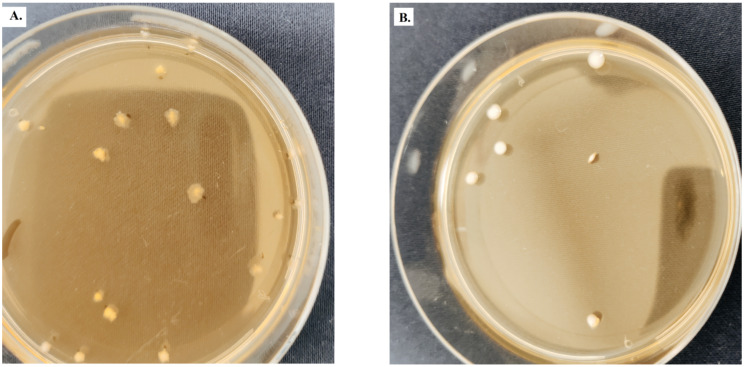
(**A**) Colonies of *L. delbrueckii* subsp. bulgaricus; (**B**) colonies of *L. casei*, *L. rhamnosus* and *L. plantarum*.

To determine the number of viable cells, the MRS agar medium was used for both the cells in the starter culture and those in the microencapsulated culture due to the different characteristics of the colonies (Figure 1). *Lactobacillus* from the starter culture forms colonies adhering to the surface of the culture medium, and they have a flat and opaque surface with crenellated edges [23]. The other colonies of *L. casei*, *L. rhamnosus* and *L. plantarum* are glossy, round and smooth, with regular edges (glossy). 

The analyses involved monitoring cell viability in the control oat beverage yogurt (starter culture) and in the synbiotic microcapsules inoculated in the oat beverage yogurt. The determination of cell viability in microcapsules was performed according to the methods described in Luca and Oroian [22] and Dimitrellou et al. [24]. A BagMixer^®^400P Lab Blender (Interlab/Interscience, Puycapel, France) was used to dissolve the microcapsule wall and release the cells; 10 g of yogurt was taken and mixed with 90 mL of 1% sodium citrate at pH 6 for 5 min in special sterile bags for BagMixer. Then, 1 mL of each type of mixture obtained (IM, I.INU, I.G, I.OLI and I.STH) was taken, and serial dilutions in 0.9% sterile saline were performed. In order to determine the number of viable cells/g of yoghurt, for each type of dilution chosen and for each type of yoghurt, inoculations were made in MRS agar plates, in triplicate. Then, they were incubated at 37 °C, under anaerobic conditions, until visible colonies were obtained (48–72 h). The total number of colonies per gram of yoghurt was calculated by multiplying the dilution factors and expressed in log10 cfu/g of yoghurt. The cell viability verification was performed after obtaining the yogurts during the storage period in refrigeration conditions once every 7 days, and then after the in vitro action for 1 h in gastric juice and for 1 h in intestinal juice.

Cell viability under the action of digestive juices was determined both at time T0 as well as during the storage period. Cell viability was monitored directly after the yogurts were obtained (T0), then it was checked for a period of 28 days at intervals of 7 days (7, 14, 21 and 28 days, namely, T7, T14, T21 and T28). We analyzed the efficacy of the encapsulated matrix formulation (2 + 2% alginate prebiotic), as described by Luca. L and Oroian M. (2021), in order to maintain high cell viability. Ten grams of each type of yogurt were placed in sterile vials with 90 mL simulated digestive juice. The vials were incubated at 37 °C for 1 h. After 1 h, the contents were transferred to sterile bags for BagMixer^®^ 400 P. Then, the same protocol described above was followed. Initially, the impact of the technological process of obtaining yogurt on cell viability was verified. Then, cell viability monitoring was performed by studying the impact of the storage time of yogurt and the action of digestive juices on the cell viability of synbiotic microcapsules. The results obtained were reported as the average of three determinations and expressed in log10 cfu/g yogurt.

### 2.7. Cell Viability Rate (R%)

The cell viability rate of synbiotic microcapsules was calculated according to Equation (1), both after the process of obtaining the finished product and during the storage period:

R = (log10N1/log10N0) × 100 [%](1)

where log10N0 represents the number of viable cells per yogurt after it was obtained, and log10N1 represents the number of viable cells existing after the yogurt storage period. The result of the equation is expressed as cfu/g [25,26].

### 2.8. Determination of pH and Total Titratable Acidity

The pH of each yogurt sample was measured using the Hach Lange HQ11D pH meter (HACH LANGE GMBH, Germany). The acidity was calculated using Equation (2), and the result was expressed in Thorner degrees (°T):

Titratable acidity = (V × 100)/V1 [°T](2)

where V—mL 0.1 N NaOH used for titration; V1—mL of yogurt taken in the analysis.

### 2.9. Color Analysis

The Konica Minolta CR400/410 colorimeter was used to determine the color in CIE L*a*b* coordinates (Konica Minolta Investment Ltd., Shanghai, China). The color differences between the microcapsule yogurt samples and the control yogurt (standard reference sample) were evaluated. Color differences were determined using Equation (3):

(3) $${\mathrm{dE}}^{*}=\sqrt{{\left({\mathrm{da}}^{*}\right)}^{2}+{\left({\mathrm{db}}^{*}\right)}^{2}+{\left({\mathrm{dL}}^{*}\right)}^{2}}$$

where dE^*^ is the total color difference, and dL^*^, da^*^ and db^*^ are the differences for L*, a*, and b* values between sample and control. Chroma C* (intensity, saturation, brightness and purity of color) was determined using Equation (4).

(4) $$C^{*}=\sqrt{{\left(a^{*}\right)}^{2}+{\left(b^{*}\right)}^{2}}$$

### 2.10. Texture Analysis

Using the TVT 6700 texturometer (Perten Instruments, Stockholm, Sweden), the yogurt texture parameters (firmness, cohesiveness, adhesiveness, elasticity and viscosity index) were analyzed. Yogurt samples (30 mm yogurt column in the bowl) were analyzed directly from the glass jar (bowl height = 60 mm; diameter = 50 mm). Three compression cycles were performed for each yogurt sample analyzed in the present study.

### 2.11. Rheological Analysis

The rheological properties of the yogurt samples were studied using the Mars 40 rheometer (Thermo Scientific, Thermo Haake, Germany), using a 2° cone–plate system (Ø 35 mm). Measurements were performed at 4 °C. To determine the linear viscoelastic region, the frequency measurements were made at 1 Hz. The frequency range was between 0.1 and 10 Hz at a tension of 1 Pa (which was in the linear viscoelastic region for all the samples). The rheological parameters surveyed were the viscoelastic parameters (G′—elastic modulus, G″—loss modulus). Experiments with controlled shear speed were performed at 100 s **^−^**^1^ at 4 °C in order to determine the viscosity.

### 2.12. Determination of Free Amino Acids

For the extraction and identification of free amino acids, 1.75 ± 0.1 g of yogurt from each sample was mixed with 15 mL of 15% trichloroacetic acid (TCA) [27]. The pH of the mixture was adjusted to 2.2 (the isoelectric precipitation point of the proteins) and the extract was further diluted to 25 mL with 15% TCA. Then, the supernatant was collected and filtered using 0.45 µm microfilters. Following this, 100 µL of filtered supernatant was analyzed for its organic components using the EZfaast GC-MS kit, following the protocol given by the manufacturer.

### 2.13. Determination of Organic Acids

To determine the organic acids, 0.5 g of each type of yogurt was distributed in plastic vials. Then, 2.5 mL of 4% metaphosphoric acid (weight/volume) was pipetted over the yoghurt. The samples were then stirred using a Vortexer homogenizer (Heathrow Scientific^®^LLC, Vernon Hills, IL, USA) and centrifuged for 5 min at 3500 rpm using a Hettich^®^ Universal 320/320R centrifuge (Andreas Hettich GmbH & Co. KG, Germany) [28]. The sample was injected into the HPLC chromatograph (Schimadzu, Kyoto, Japan). The separation was performed on a Phenomenex Kinetex^®^ 5 μm C18 100 Å 250 × 4.6 mm HPLC column. A mixture of 0.5% metaphosphoric acid and acetonitrile was used as the mobile phase at a flow rate of 0.8 mL/min. The injection volume was 10 μL. Organic acids were identified by comparing retention times and the peak area standards of lactic acid, gluconic acid, acetic acid, propionic acid and butyric acid. The organic acid content was expressed in mg/L.

### 2.14. Sensory Analyses

The 5 types of oat yogurt were subjected to sensory analysis for each moment of storage. The sensory evaluation of yogurts was performed by 63 semi-trained panelists. The group consisted of students, professors and staff from the Faculty of Food Engineering (“Stefan cel Mare”, University of Suceava, Suceava, Romania). A standard method of evaluation, namely, the scoring method, was used to evaluate the sensory analysis of fermented plant products. For each characteristic (appearance, color and consistency; smell; taste), 9 steps were established (from 0 to 9), 0 being the weakest and 9 the strongest expression of the characteristic. Yogurt samples were presented in glass jars for easier assessment of the “appearance, color and consistency” characteristic. For each characteristic, the mean score (Pm) and the weighted average score (Pmp) were calculated using Equation (5):

(5) $$P_{\mathrm{mp}}={\;P}_{m}\cdot f_{p}\;$$

where f_p_ is the factor (appearance, color and consistency = 1.5; smell = 0.5; taste = 2). The evaluation of the organoleptic quality of the product was obtained based on the average score given by the tasters in comparison with a scale of 36 points. The yogurt samples were subjected to organoleptic analysis, and also to the analysis of the sensory characteristic: oat flavor. In order to more easily recognize the presence or absence of oat aroma, a sample of oatmeal was offered for tasting. Thus, 9 steps were also established, from 0 to 9, where 9 represents the fact that the oat aroma felt “intense” and 0 was “not at all”.

### 2.15. Statistical Analysis

Statistical analysis was performed using XLSTAT software trial version (Microsoft, New York, NY, USA). The results obtained were reported as the average of three determinations. Statistical differences between groups were determined by a single analysis of variance (ANOVA), followed by Tukey test (HSD) to test whether the relationship between the two data sets is statistically significant, Fisher (LSD) to compare the variants and Levene to determine the degree of similarity between the dependent and independent variables. Principal component analysis (PCA) was used to interpret the results obtained in the present study.

## 3. Results and Discussion

### 3.1. Viability Study of Probiotic Strains in Oat Milk Yogurt

**Figure 2:**
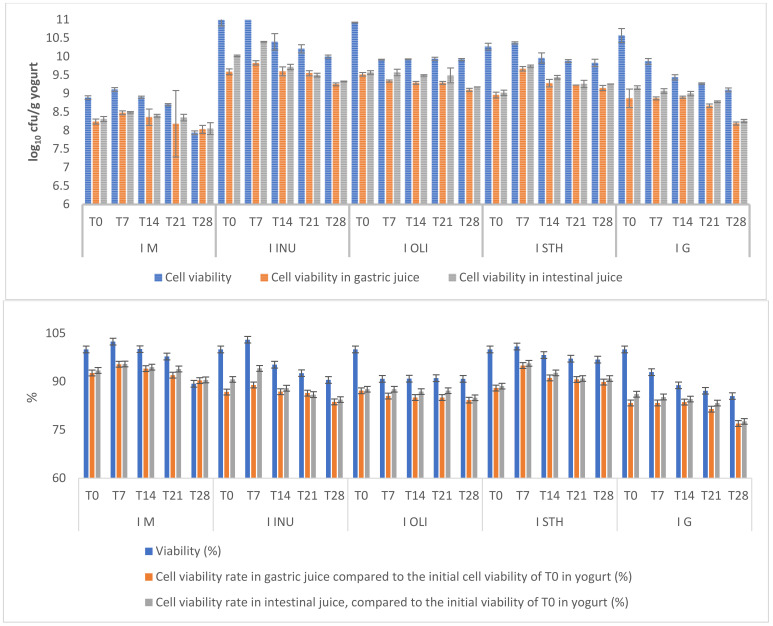
Cell viability of oat milk yogurt samples. I M—yogurt without synbiotic microcapsules (control sample); I STH—yogurt containing synbiotic microcapsules with starch; I INU—yogurt containing synbiotic microcapsules with inulin; I G—yogurt containing microcapsules with glucose; I OLI—yogurt containing synbiotic microcapsules with oligofructose. T0 = the moment of analysis from the day of obtaining the yogurt; –T7, –T14, –T21 and –T28—yogurt analysis moments reported relative to day T0. Each bar presents the mean of three replicates ± standard deviation.

In the case of oat yogurt enriched with synbiotic microcapsules, the most important parameter to be determined is the number of viable probiotic cells. After stopping the yoghurt fermentation process after 16 h of incubation at 43 °C through storage in refrigeration conditions, the yogurt was then subjected to microbiological analyses in order to verify the cell viability of the synbiotic microcapsules. After obtaining the yogurts, the viability of the microencapsulated cells inside the yogurt and the cell viability of the starter culture present in the yogurt were monitored. The effect of prebiotics (INU, OLI and STH) and also of glucose (G) in the matrix of microcapsules in oat yogurt on cell viability was monitored both at the time of obtaining the yogurt and during the 28 days of storage at 4 °C. The results obtained after performing the experiments showed that the cell viability of the synbiotic microcapsules in the four types of yogurts was over 10 log10 cfu/g (Figure 2).

These results indicate that during the technological process of obtaining the food product, the encapsulation matrices provided protection to the probiotic cells during the 28 days. In the control sample, the viability of the cells in the starter culture was maintained above the limits recommended by the FAO/WHO (10^6^–10^7^ cfu/g) during storage. The experimental data obtained from the analysis of the cellular viability of microcapsules in yoghurts at the time T0 showed significant differences (*p* < 0.05) for yogurts I.INU vs. I.STH and I.OLI vs. I.STH. No significant differences were observed in the rest of the analyzed samples (*p* > 0.05) (Figure 2).

The experimental data obtained after 7, 14, 21 and 28 days of storage were reported at the time T0 in order to verify the cell viability maintenance rates. After the first 7 days of storage, it was found that the rates of maintaining cell viability in the synbiotic microcapsules in I.INU yogurt and those in I.STH yogurt showed the highest values. The viability rate of the cells compared to T0 for I.INU was 103.03 ± 0.04%, and for I.STH was 100.90 ± 0.07%. For the other two types of yoghurt with microcapsules, after 7 days of storage (T7), the cell viability rate was 92.93 ± 0.33% for probiotic cells in I.G microcapsules and 90.84 ± 0.03% for those from the microcapsules in I.OLI (Figure 2). After 14 and 28 days of storage, a decrease in the cell viability rate of microencapsulated probiotic strains was observed. After 28 days of storage (T28), the lowest rate of cell viability was recorded for glucose microcapsules (85.52 ± 0.04%), due to its simple chemical structure compared to the structure of the others. The rate of cell viability in other types of microcapsules depended on the complexity of the chemical structure of the carbon source. Thus, for synbiotic microcapsules with oligofructose in the capsule matrix, cell viability was 9.92 ± 0.03 log10 cfu/g yogurt with a survival rate of 90.84 ± 0.01%, followed by synbiotic microcapsules with starch at 9.83 ± 0.10 log10 cfu/g (96.86 ± 0.08%), and then those with inulin at 10.00 ± 0.04 log10 cfu/g (90.52 ± 0.01%). The cell viability rate in oat yogurt without microcapsules was over 100%, even after 14 days of storage, which indicates that yogurt is a source of nutrition for starter cells. After 28 days, the number of viable cells in the starter culture was 7.94 ± 0.05 log10 cfu/g, with a survival rate of 89.34 ± 0.01% compared to T0.

### 3.2. The Viability of the Probiotics Strains on Simulated In Vitro Gastric Juice

In order for the consumer to benefit from the beneficial effects of probiotics, it is very important that the food contains a sufficient number of viable cells (10^6^–10^7^ cfu/g) [29] when it reaches the intestine. Therefore, probiotics must survive the passage through the gastrointestinal tract. Cell viability decreases under the action of stomach acid and bile salts in the intestinal tract [30].

The experimental data obtained from the action of gastric juice and intestinal juice on probiotic strains from the four types of yogurts with microcapsules (Figure 2) showed that at the time T0 the cell viability rate was over 80%. After the action of simulated gastric juice in vitro, the lowest rate of cell viability was recorded in glucose microcapsules (83.36 ± 0.15%), with a cell viability of 8.87 ± 0.25 log10 cfu/g yogurt. This cell viability rate was maintained after 7 days and 14 days of storage, respectively, without any significant differences (*p* > 0.05) compared to T0. However, significant differences were observed starting with the 21st day of storage (*p* < 0.05). Similar changes occurred under the action of intestinal juice at the time T0 when a cell viability rate of 86.09 ± 0.03% and a viable cell count of 9.16 ± 0.05 log10 cfu/g yoghurt was recorded. At T7 for the microcapsules with glucose from I G, the determined cell viability rate was 85.24 ± 0.05%, with a viable cell count of 9.07 ± 0.06 log10 cfu/g.

After the glucose microcapsules, the highest cell viability rate was determined for oligofructose synbiotic microcapsules in the I.OLI yogurt. Therefore, following the action of gastric juice at T0, a viable cell count of 9.52 ± 0.05 log10 cfu/g yogurt was obtained and at T7, a survival rate of 85.53 ± 0.10% was recorded, with a viable cell count of 9.34 ± 0.03 log10 cfu/g yogurt. After the action of intestinal juice, at T0, a survival rate of 87.18 ± 0.02% was recorded, with a viable cell count of 9.57 ± 0.05 log10 cfu/g yogurt, and at the time T7, a survival rate of 87.64 ± 0.03% and a viable cell count of 9.57 ± 0.09 log10 cfu/g yoghurt were recorded. Prior to the action of digestive juices, the rate of cell viability and the number of viable cells in the microcapsules with INU and STH from T7 had higher values than those obtained at T0 in the case of microcapsules subjected to the action of the two digestive juices. Significant differences were recorded between all values obtained (*p* < 0.05). The experimental data obtained from the verification of cell viability after 28 days of storage showed that all values were within the limits recommended by the FAO/WHO (2003). Thus, the number of viable cells recorded after the storage period in ascending order are: 8.03 ± 0.11 log10 cfu/g, I.M in gastric juice; 8.05 ± 0.16 log10 cfu/g, I.M in intestinal juice; 8.19 ± 0.04 log10 cfu/g, IG in gastric juice and 8.26 ± 0.04 log10 cfu/g, IG in intestinal juice; 9.10 ± 0.04 log10 cfu/g, I OLI in gastric juice and 9.18 ± 0.01 log10 cfu/g, I OLI in intestinal juice; 9.15 ± 0.07 log10 cfu/g, I STH in gastric juice and 9.26 ± 0.01 log10 cfu/g, I STH in intestinal juice; 9.25 ± 0.04 log10 cfu/g, I INU in gastric juice and 9.33 ± 0.01 log10 cfu/g, I INU in intestinal juice. Significant differences were found between all determined values (*p* < 0.05). The results obtained, such as cell viability above the minimum limit recommended by the FAO, indicated the fact that oats are an optimal substrate for the growth of probiotic strains. This is due to the presence of indigestible components that can also be used as prebiotics [31].

### 3.3. Physico-Chemical Properties of Oat Yogurt

#### 3.3.1. Color

The values of the color parameters (L*, a* and b*) obtained for all yogurt samples can be found in Table 2. The only significant differences for the value of a* can be observed at the moment T0 for sample I.STH compared to I.M, and in I.INU, the value of b* showed different values from those of sample I.M. In terms of brightness (L*), significant differences were observed between T0 and T28 for all yogurt samples by comparing the values obtained with sample I.M, except sample I.STH. The experimental data showed that the addition of synbiotic microcapsules produced changes in the color of yogurts compared to I.M. However, these differences are not detected by the human eye because the value of dE* < 3 [15]. The color differences (dE*) are presented in Table 3. At the time T0, the yogurt samples that have the values closest to those of sample I.M were I.INU (dL* = 0.03) and I.STH (dL* = −0.02) for the parameter dL.

#### 3.3.2. Study of the Evolution of Titratable Acidity and pH

After 16 h of fermentation, the pH values varied significantly for all yogurt samples compared to the I.M sample (*p* < 0.001). The values ranged between 3.53 ± 0.01 and 3.82 ± 0.01. The lowest pH value was obtained in the I.INU yogurt sample. Of all the yogurt samples analyzed, it was observed that the lowest pH values were obtained at T28 (3.55 ± 0.10). After 14 days of storage, the I.G samples recorded the highest pH values compared to the I.M sample (T0—3.83 ± 0.00; T7—3.72 ± 0.00 and T14—3.77 ± 0.00), and after 21 days of storage, the pH dropped to 3.58. After 28 days, the pH value for I.G was 3.49 ± 0.00. The experimental data for yogurt samples with synbiotic microcapsules with starch and oligofructose showed a stability of pH values at T7 and T14, and then they decreased by 0.06 for I.OLI and 0.07 for I.STH. After 28 days of storage, the pH value was 3.52 ± 0.00 for I.OLI and 3.55 ± 0.00 for I.STH.

According to the data obtained, the presence of synbiotic microcapsules, and also the composition of their matrices, influenced the pH of yogurts as a result of the viability of the cells in the starter culture throughout storage. During storage, the content of organic acids increased due to cellular metabolism, a phenomenon possible due to the presence of additional carbon sources in the microcapsules in the composition of the yogurts.

Luana et al. [32] obtained a fermented drink based on oatmeal after 8 and 12 h of fermentation with the help of different strains of lactobacilli: *L. plantarum* LP01, LP06, LP09, LP32, LP39, LP40 and LP48; L casei LC10, LC11 and LC03; and L. *paracasei* LPC02, LPC16. After the fermentation period, the pH of the drinks obtained by Luana et al. [32] was higher than that obtained in the present study (mean pH value 4.2). These values were influenced by the fermentation duration and the type of starter cultures used.

Mårtensson et al. [14], in their study, used a frozen oatmeal concentrate subjected to fermentation for 16 h to obtain a fermented beverage. In order to obtain this fermented beverage, two different types of cultures were used: *Streptococcus* salivarius subsp. *thermophilus* and L. *delbrueckii* subsp. *bulgaricus* (Visby Tonder A/S Danemarca), and *Streptococcus salivarius* subsp. *thermophilus*, L. acidophillus and *Bifidobacterium* spp. (Chr. Hansen Danemarca). Therefore, Mårtensson et al. [14] reported pH values between 3.8 and 4.0 and concluded that a low pH value of an oat-based fermented product, similar to classic yogurt, considerably increases the overall acceptability for this product.

In the study performed by Gupta et al. [33], the pH of an oat-based beverage fermented for 8 h with L. plantarum ATCC 8014, after 21 days of storage, was 4.0. The results obtained by Bernat et al., (2015) [14] for a fermented oat-based beverage with a mixed culture consisting of L. reuteri ATCC 55,730 and S. thermophilus CECT 986 showed a pH value, after 21 days of storage, of 3.61, and after 28 days, the pH value was 3.30. According to them, during fermentation, the temperature and type of strains used influenced the acidity of the cereal-based beverages. Helland et al. [34] reported a decrease in pH values (3.4–4.4) in cereal-based products after 21 days of storage. In the present study, after the 28 days of storage, the pH values obtained for the yogurt samples were higher than 3.4.

In the studied yogurt samples, an increase in titratable acidity was observed during the storage period (Table 2). Thus, after 28 days of storage, the titratable acidity compared to the moment T0 increased by 58.95% in I.M yogurt, by 44.12% in I.INU yogurt, by 44.91% in I.OLI yogurt, by 41.30% in I.STH yogurt and by 40.10% in I.G yogurt. The I.M yogurt sample had the lowest titratable acidity (0.18 ± 0.01% lactic acid), followed by that of I.INU yogurt (0.24 ± 0.01% lactic acid), I.STH (0.25 ± 0.02% lactic acid), IG (0.26 ± 0.01% lactic acid) and the last being that of I.OLI yogurt (0.27 ± 0.01% lactic acid). Luana et al. [32] obtained titratable acidity values between 13 °T and 32 °T for a fermented oat drink after 8 h of fermentation. Mårtensson et al. [14], after 16 h of oat-based beverage fermentation, obtained titratable acidity values between 0.2% and 0.5% depending on the strains used, and Bernat et al. [15] obtained values of 0.16 g lactic acid × 100 mL^−1^ immediately after the fermentation process and, after 28 days, the value of titratable acidity was 0.50 g lactic acid × 100 mL^−1^. Given the experimental results of this study, confirmed by the results obtained by other researchers with various fermented beverages, it was found that the presence of synbiotic microcapsules did not produce modification during the technological flow of oat yogurt production.

#### 3.3.3. Study of the Influence of Synbiotic Microcapsules on the Texture of Yogurt from Oats

The sensory properties of yogurt, such as texture, characteristic odor and taste, are indicators of acceptability for consumers. Texture is a parameter that can only be influenced by the production process, as opposed to smell and taste, which can be modified or adapted later [35]. In the present study, a quantity of 16% dry matter (*w*/*v*) was required to obtain an oat beverage yoghurt with a texture similar to that of animal milk. Creaminess, viscosity and consistency have been improved with increasing vegetable fat concentrations (coconut oil 1.5%). Therefore, after stopping the fermentation process, the experimental data showed that the presence of microcapsules from the four types of yogurts produced had a minor influence on the texture compared to the control sample I.M. Thus, for the I.M yogurt samples, the value for hardness was 208.13 ± 4.01 g, and in the other samples, this value was lower (Table 2), and the lowest value was for the I.INU yogurt (181.00 ± 2.01), with the ranking as follows: 134.00 ± 1.00 g for I.STH yogurt; 127.33 ± 2.60 g for I.INU yogurt; 122.33 ± 1.20 g for yogurt I.G; 117.00 ± 1.87 g for I.OLI yogurt. After the storage period (T28), the values obtained for hardness increased compared to T0. The experimental results obtained for cohesiveness showed that the values of this characteristic decreased from T0 to T28 for all yogurt samples. The highest value of cohesiveness after 28 days was obtained for the I.G yogurt sample (1.26 ± 0.38), and the lowest value was obtained for the I.STH yogurt sample (0.84 ± 0.20). For the other samples, the values of cohesiveness at the time T28 were as follows: 0.95 ± 0.15 for the control yogurt sample; 1.02 ± 0.26 for I.OLI and 1.14 ± 0.28 for I.INU. By comparing the data obtained for adhesiveness, from T0 to T28, we can see an increase in values compared to T0.

#### 3.3.4. Rheological Properties of Oat Yogurt

**Figure 3:**
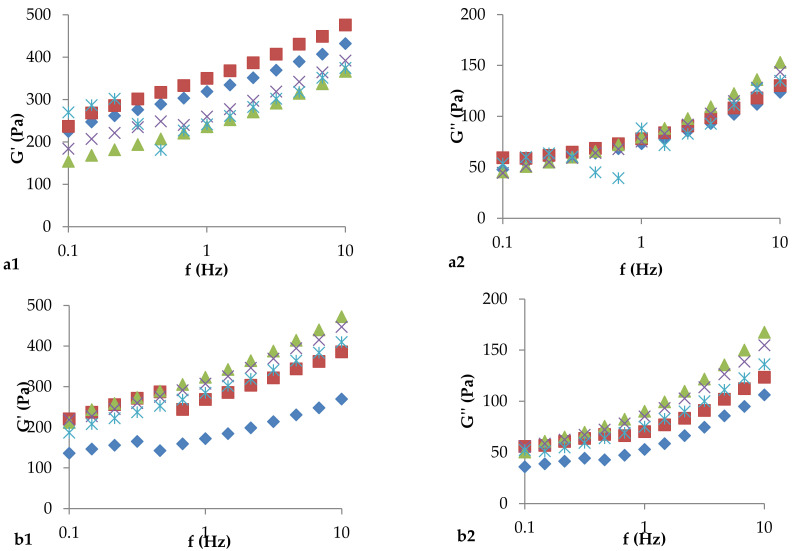

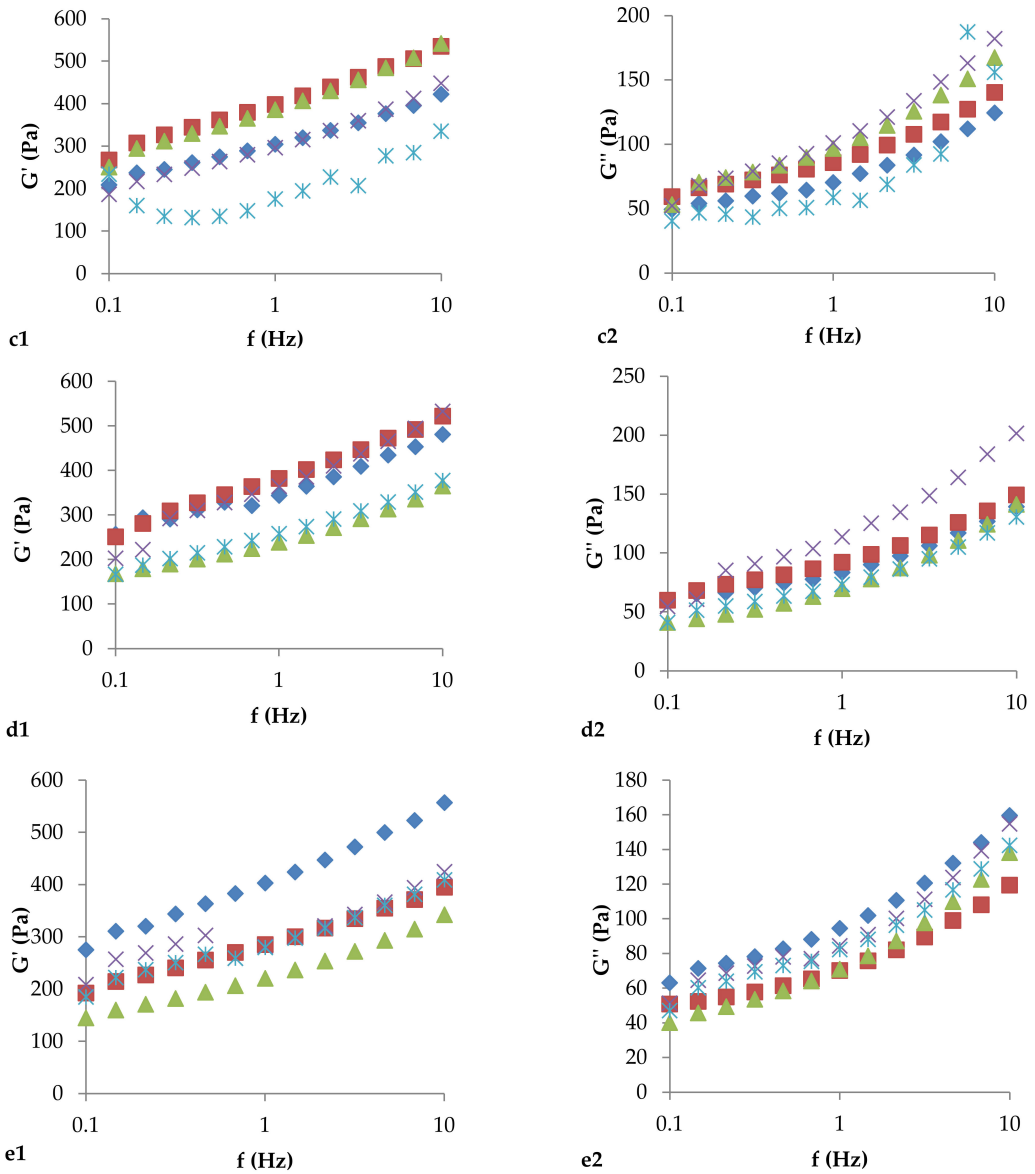
Evolution of viscoelastic parameters during the storage period of vegetable yogurt: (1) G′—modulus of elasticity, (2) G″—modulus of viscosity; (**a**)—control yogurt, without synbiotic microcapsules, (**b**)—yogurt containing microcapsules with glucose, (**c**)—yogurt containing synbiotic microcapsules with starch, (**d**)—yogurt containing synbiotic microcapsules with oligofructose, (**e**)—yogurt containing synbiotic microcapsules with inulin; 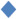 T0—the moment of analysis from the day of obtaining the yogurt; 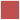 T7, 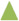 T14, 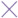 T21 and 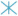 T28—yogurt analysis moments relative to day T0.

Rheological parameters play a key role in defining the textural and sensory perception of a product. The rheological properties provide the most important information related to the broader properties of yogurt. It was observed that, in relation to the control sample, the viscosity values of the yogurt samples, during the storage period, were influenced by the presence of microcapsules and their composition. At the same time, there was an increase in viscosity values until the 14th day, and then there was a gradual decrease until the last day of storage. According to the values obtained for viscosity on day 28, it was observed that they do not change considerably compared to those obtained in T0. The variation in the viscosity of the yogurt samples was probably due to the protein hydrolysis performed by the starter culture, but it was also due to the presence of β-glucan fibers in the oats. It may be hypothesized that the culture starter YC-350 hydrolyzed this compound to obtain the nutrients needed to grow and maintain cell viability. The β-glucans have the ability to increase the viscosity of aqueous solutions [14]; therefore, in oats, β-glucans influenced the thickening capacity of yogurt. In addition to β-glucans, the exopolysaccharides synthesized by the cells of the starter culture, and also those inside the different types of microcapsules, contribute to the increase in the viscosity of yogurt [36]. At the same time, the obtained results indicated a typical behavior for non-Newtonian fluids because, with the increase in the shear rate, there is a decrease in the values for viscosity. The evolution of the viscoelastic parameters during the storage period is shown in Figure 3. The viscoelastic properties of the samples and their frequency dependence indicated a nonlinear behavior of all yogurt samples. Nonlinear behavior occurs as a result of changes in the structure of yogurt, in which macroscopic particles interacted downward with the dispersion medium due to disturbances in the increasing shear rate. The results indicated a predominantly elastic behavior, considering the relationship between the modulus of elasticity and the modulus of viscosity (G′ > G″) for all yogurt samples during the storage period. Consequently, all samples can be described as soft fluid gels.

### 3.4. Sensory Analysis

**Figure 4:**
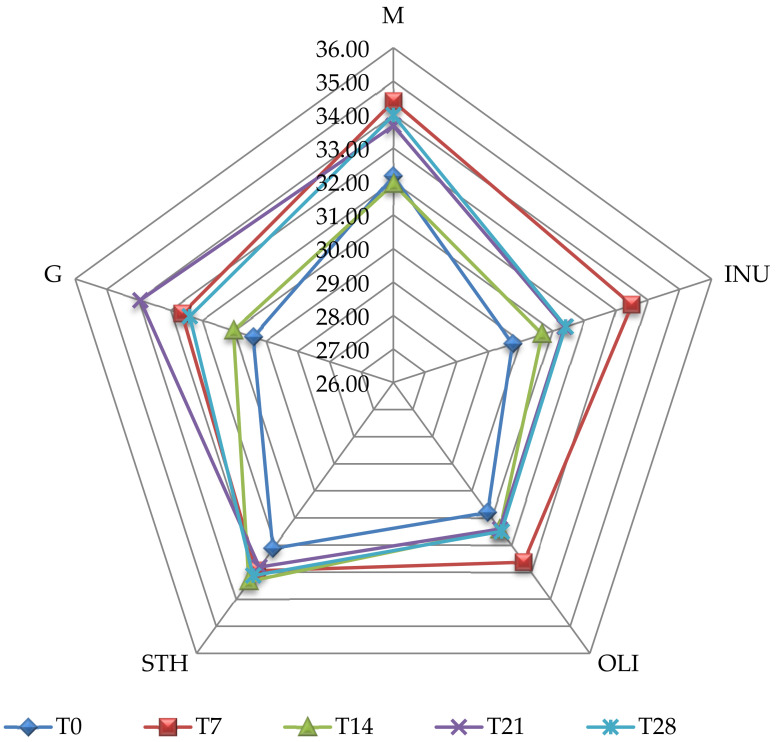
Sensory analysis of yogurt samples. Pmp—general acceptability; M—control yogurt, without synbiotic microcapsules; INU—yogurt containing synbiotic microcapsules with inulin; OLI—yogurt containing synbiotic microcapsules with oligofructose; STH—yogurt containing synbiotic microcapsules with starch; G—yogurt containing microcapsules with glucose; T0 = the moment of analysis from the day of obtaining the yogurt; –T7, –T14, –T21 and –T28—yogurt analysis moments reported relative to day T0.

In terms of sensory properties, oats have a low acceptability among consumers. This low acceptability is mainly due to characteristics such as taste of earth, cereals, etc. [15,32]. The results of the sensory analysis showed that the oat flavor was perceived as weak in most yogurt samples, except for the I.STH yogurt which, in T14, had a flavor of medium intensity (Table 2). After 28 days of storage, the aroma was perceived with significant differences between the yoghurts (*p* < 0.05); so, if at T0, the mean score was 2.91 ± 0.23 for I.M, in T28, it increased to 2.95 ± 0.28. The same situation was for the I G sample. In contrast, for the I.OLI and I.INU samples, the values increased significantly (*p* < 0.05), which indicates that the microcapsule matrix enhanced the initial flavor. For sample I.STH, the oat flavor was maintained at average throughout the monitoring due to the composition of the microcapsules (Table 2). I.OLI samples showed the lowest values in terms of oat flavor, and the most intense oat flavor was perceived by tasters in samples I.STH. At T14, the most intense oat flavor (2.78 ± 0.24) was reported, and on the last day of storage (T28), the value obtained for this parameter was 3.05 ± 0.28. To establish the organoleptic qualities for each type of yogurt, the analysis of the weighted average score (Pmp) was performed. The data obtained are presented in Figure 4. The results of the sensory evaluation from T0 show that the yogurt samples are acceptable.

The rating obtained by the group of tasters was “good” for all samples. Statistically, this rating showed significant differences between samples. The results of the analysis of the sensory profile showed that as a result of the fermentation process, the oat aroma decreased, and the products showed similar characteristics to a regular yogurt (consistency, taste and smell). The group of tasters classified the products into two types, good and very good, giving the qualifier “good” for I.G, I.OLI and I.INU and “very good” for I.M and I.STH.

### 3.5. Study of Organic Acid Content

**Figure 5:**
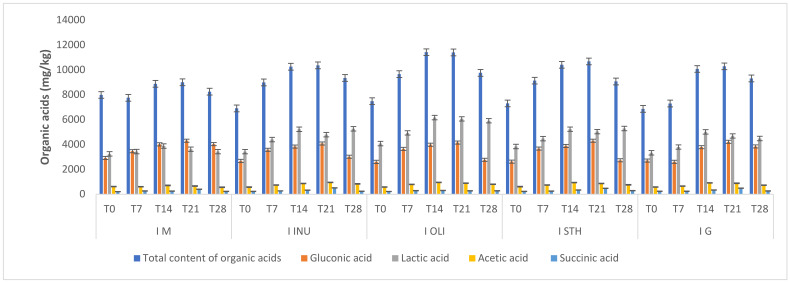
Profile of organic acids in oat milk yogurt samples. I M—yogurt without synbiotic microcapsules (control sample); I STH—yogurt containing synbiotic microcapsules with starch; I INU—yogurt containing synbiotic microcapsules with inulin; I G—yogurt containing microcapsules with glucose; I OLI—yogurt containing synbiotic microcapsules with oligofructose. T0 = the moment of analysis from the day of obtaining the yogurt; –T7, –T14, –T21 and –T28—yogurt analysis moments reported relative to day T0. Each bar presents the mean of three replicates ± standard deviation.

Determining the content of organic acids in food is an important parameter due to its interference with the quality, stability and nutritional profile of the finished product [37]. This analysis of organic acids was carried out in order to verify the quality of the yogurt samples and their shelf life. At the same time, we verified the influence of microcapsules on the content of organic acids in the yogurt samples with microcapsules compared to the control sample (Figure 5). Both *Streptococcus thermophilus* and the lactobacilli present in the yogurt samples are lactic acid-producing bacteria. Lactic bacteria have complex nutritional requirements for fermentable carbohydrates, amino acids, nucleic acids or other substrates, but they can also obtain their metabolic energy from the homofermentative or heterofermentative fermentation of carbohydrates [38]. As a result of these fermentation processes, in the yogurt obtained from oat beverages, we identified organic acids: lactic, gluconic, citric and succinic. After 7 days of storage, an increase in the organic acid content (8560.73 ± 994.63 mg/kg) was observed due to the cellular metabolism, both at the level of the microcapsules and at the level of the food matrix. This increase took place until T21 (10,340.27 ± 869.90 mg/kg) for all types of yogurts, followed by a decrease at the time T28 (9141.75 ± 561.92 mg/kg) following a possible depletion of metabolic resources. The increase in organic acid content was influenced by the presence of microcapsules that provided additional resources for organic acid biosynthesis. The experimental data showed that the predominant organic acid is lactic acid (the maximum value recorded was for I.INU, at 5426.17 ± 902.42 mg/kg), followed by gluconic acid, acetic acid and succinic acid. At the same time, the lack of formic, propionic and butyric acids was found. Their absence is beneficial and indicates the fact that a food product without unwanted fermentations can be obtained. Lactic, gluconic and acetic acids are the main compounds that improve the flavor in fermented cereal products [39], and this aspect was confirmed by the sensory analysis of yogurts. The production of lactic and acetic acids is attributed to homo- and heterofermentative lactobacilli from yogurt capable of metabolizing disaccharides from oat substrates, such as maltose, sucrose, oligofructose and starch [40]. At the same time, these organic compounds also provided a high stability of yogurt, demonstrating their role as a food preservative.

The production of gluconic acid occurs as a result of the metabolism of sugar added to the composition of yogurt. This type of organic acid is the result of glucose oxidation under the action of glucosoxidase, and it also helps to improve the flavor of yogurt because it gives a sweet–bitter taste [39]. Succinic acid has made an important contribution to improving the flavor of yogurts, and it is generally used as an additive in processed foods. The presence of these acids explains the fact that the oat flavor identified in the boiled oat sample was modified as a result of the fermentation processes, an aspect confirmed by the results obtained from the sensory analysis.

### 3.6. Study of Amino Acid Content

**Figure 6:**
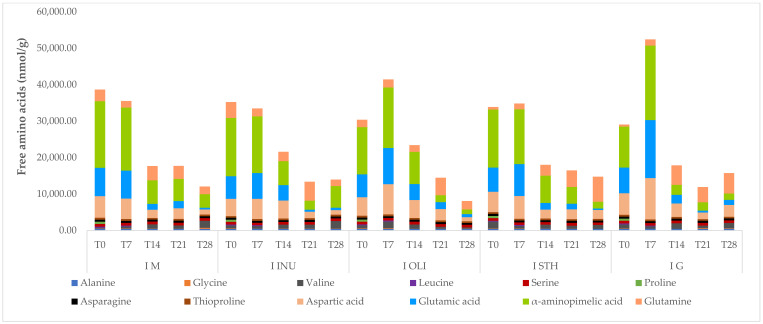
Amino acid profile of oat milk yogurt samples. I M—yogurt without synbiotic microcapsules (control sample); I STH—yogurt containing synbiotic microcapsules with starch; I INU—yogurt containing synbiotic microcapsules with inulin; I G—yogurt containing microcapsules with glucose; I OLI—yogurt containing synbiotic microcapsules with oligofructose. T0 = the moment of analysis from the day of obtaining the yogurt; –T7, –T14, –T21 and –T28—yogurt analysis moments reported relative to day T0.

For an ideal dietary balance, it is necessary that the food consumed has a high nutritional value. This represents the quality of food and demonstrates its ability to meet the nutritional needs of the human body in terms of protein, carbohydrates and vitamins. The higher this value, the more the product can provide the proper nutritional requirements for a balanced diet. The most important aspect of a food, from a nutritional point of view, is the content of essential amino acids, because they contain carbon skeletons that cannot be synthesized by the human body and which, therefore, must be provided from the diet. For this reason, essential amino acids are more important for increasing and maintaining metabolic needs than non-essential ones. Compared to other cereals, oats have a higher content of proteins, but also of essential amino acids, which gives them a high nutritional value. The amino acid content present in oats is very close to the optimal composition for the human body [35]. It was found that this amino acid profile present in oat grains helped maintain a high cell viability for the starter culture throughout the storage period of yogurts, after the completion of the oat vegetable beverage fermentation process. Following the research, the data obtained showed that the five types of oat milk yogurt have, in their composition, the essential amino acids glycine, valine, leucine and glutamine, and also the non-essential amino acids alanine, serine, proline, asparagine, thioproline, aspartic acid, glutamic acid and α-aminopimelic acid. The results of the amino acid profile after the 28 days of yogurt storage (Figure 6) indicated a decrease compared to the time T0. It was observed that for I.M, the total amount of amino acids in T0 was 38,668 nmol/g, and after 28 days, the total amount of amino acids was reduced by 31% (12,044 nmol/g). For I.G, we registered in T0 a total amount of amino acids of 29,055 nmol/g, and in T28, an amount of 15,757 nmol/g. 

The experimental data showed that two of the free amino acids, proline and leucine, were present in yogurt samples only in the first days after obtaining them. Thus, the amino acid proline was present in all types of yogurts only on the day they were obtained, possibly due to the fact that it was used as a source of nitrogen by the microorganisms present in the yogurts. The same observation was made for the amino acid leucine, which was present in the yogurts only in the first 7 days since obtaining them, and then probably was used in metabolic activity by microorganisms present in yogurts due to the increased requirement of nitrogen.

### 3.7. Principal Component Analysis

**Figure 7:**
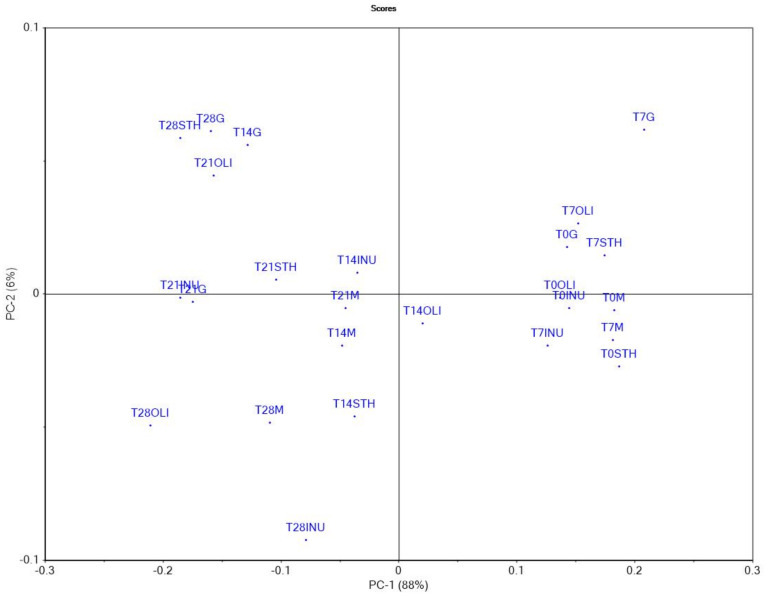
Analysis of the main components—scores: T_0_–T_28_ M—yogurt control sample, analyzed from the moment of obtaining it to day 28 (T_28_); T0-T_28_ INU—yogurt sample with synbiotic microcapsules with inulin, analyzed from the moment of obtaining it to day 28 (T_28_); T0 OLI—yogurt sample with synbiotic microcapsules with oligofructose, analyzed from the moment of obtaining it to day 28 (T_28_); T0 STH—yogurt sample with synbiotic microcapsules with starch, analyzed from the moment of obtaining it to day 28 (T_28_); T0 G—yogurt sample with glucose microcapsules, analyzed from the moment of obtaining it to day 28 (T_28_).

The results of the determinations obtained for a group of samples can be compared using principal component analysis (PCA). In the present study, this statistical method was used to analyze and identify the samples of oat milk yogurt with similar characteristics. The first major component (PC-1) explained 89% of the variation, while the second major component (PC-2) explained 5% of the variation; together, the first two main components explained 94% of the initial variability. The separation of yogurt samples from oat milk was performed according to the addition of synbiotic microcapsules and analysis times. As shown in Figure 7, the samples analyzed in T0 are grouped in a single quadrant. Among the samples analyzed in T0, three samples analyzed on day 7 (I.M, I.STH and I.INU) are also interspersed, which shows that the I.M sample, the I.STH sample and the I.INU sample showed, after 7 days of storage, values of the analyzed parameters close to those results at the time T0.

**Figure 8:**
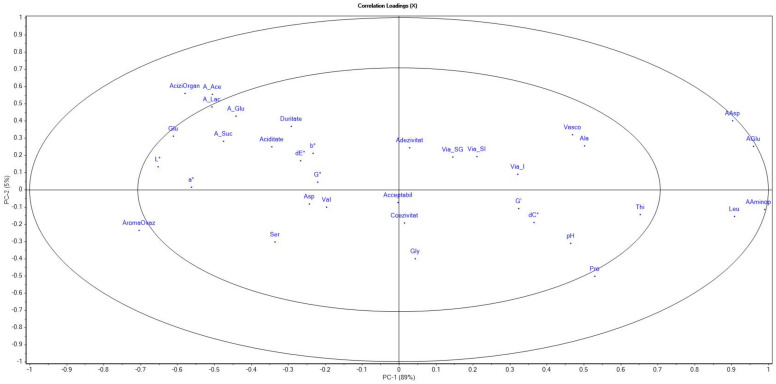
Analysis of the main components—influence of the parameters: pH; L*, a*, b*, dE*—total color difference; dC*—opaque/brightness difference; Acidity—titratable acidity; AciziOrgan—organic acids; A_Glu—gluconic acid; A_Lac—lactic acid; A_Ace—acetic acid; A_Suc—succinic acid; Acceptable—acceptability; Adhesiveness—adhesiveness; Cohesiveness—cohesiveness; Via_SG—cell viability in gastric juice; Via_SI—cell viability in intestinal juice; Via—cell viability in yogurt; Vasco—viscoelasticity; Duritate—hardness; adezivit—adhesivity; AromaOvaz—oat aroma; Vasco—viscosity; Aciditate—acidity; G′—elastic mode; G″—viscous mode; AAminop—α-aminopimelic acid; Ser—serine; Pro—proline; Asp—asparagine; Thi—thioproline; AAsp—aspartic acid; AGlu—glutamic acid; Ala—alanine; Gly—glycine; Val—valine; Leu—leucine; Glu—glutamine.

All yogurt samples with the addition of synbiotic microcapsules, analyzed after 21 days, could be grouped with similar values of the analyzed parameters. In the same quadrant, two of the samples analyzed in T28 are present (I.G and I.STH). The other three samples analyzed after 28 days (I.OLI, I.INU and I.M) were separated in another quadrant. The samples analyzed after 14 days from the time of obtaining the yogurts could not be clearly separated. As can be seen in Figure 8, the parameters located in the outer ellipse have a greater contribution to variability than the parameters located in the inner ellipse. Adhesiveness, cohesiveness and acceptability did not have a significant influence because they are located near point 0.

## 4. Conclusions

In this paper, a food matrix made of an oat beverage for three types of synbiotic microcapsules for 28 days was studied in order to develop a new functional food. Probiotic microorganisms are used in food to improve their nutritional properties. To achieve this, the probiotics must survive the passage through the gastrointestinal tract. The actions of stomach acid and bile salts in the intestinal tract are the main factors that decrease cell viability in ingested food. The experimental data showed the maintenance of an optimal cell viability in the food throughout its storage period. Additionally, after the action of simulated gastrointestinal juices in vitro, the viability of probiotic cells was verified, and it was proved that the encapsulation matrices (INU, OLI, STH and G) ensured the presence in the product of a number of recommended viable cells after 28 days of storage. The highest cell viability was found in the sample with the addition of inulin synbiotic microcapsules, followed by the I.STH sample and the I.OLI sample. Maintaining the viability of probiotic cells in functional foods is important because it plays an important role in ensuring the health benefits of consumers. The results of the sensory evaluation showed that oat yoghurts, with and without synbiotic microcapsules, were appreciated throughout the storage period with the qualifier “good” and “very good”. The analysis of the amino acid content completed the study of oat milk yogurt samples by providing the nutritional profile of the finished product. Following the analysis of the amino acid content, 12 amino acids were identified, of which 4 were essential amino acids and 8 were non-essential amino acids. In conclusion, microencapsulated probiotic strains can be added to fermented non-dairy products, such as vegetable yoghurt from oat milk. All data indicate that a functional food product has been obtained that can provide consumers with health benefits as a result of an optimal intake of probiotic cells.

## Figures and Tables

**Table 1:** The size and cell load of the microcapsules used.

Microcapsules Type with 2% Alginate	Dimension (μm)	Cell Load (log_10_ cfu/g)
2% glucose	215.00–404.00	11.90 ± 0.16
2% oligofructose	203.00–474.00	12.03 ± 0.20
2% starch	230.00–495.00	12.53 ± 0.05
2% inulin	211.00–482.00	12.32 ± 0.09

**Table 2:** Physico-chemical and texture parameters of oat milk yogurt samples. Mean values and standard deviations.

Parameter	Storage Period (Days)					Value F	Type of Yogurt					Value F
	0	7	14	21	28		I M	I G	I INU	I OLI	I STH	
L *	74.98 ± 0.02 ^ab^	74.64 ± 0.01 ^ab^	73.47 ± 0.03 ^a^	76.09 ± 0.01 ^b^	76.67 ± 0.02 ^b^	4.08 *	74.98 ± 0.02 ^a^	74.37 ± 0.02 ^a^	75.44 ± 0.01 ^a^	75.39 ± 0.01 ^a^	75.67 ± 0.01 ^a^	0.57 ns
a *	−4.96 ± 0.01 ^a^	−5.01 ± 0.02 ^a^	−4.69 ± 0.03 ^b^	−4.50 ± 0.01 ^c^	−4.95 ± 0.02 ^a^	59.78 ***	−4.76 ± 0.01 ^a^	−4.88 ± 0.02 ^a^	−4.83 ± 0.05 ^a^	−4.85 ± 0.05 ^a^	−4.79 ± 0.06 ^a^	0.72 ns
b *	14.39 ± 0.02 ^b^	14.46 ± 0.02 ^b^	15.57 ± 0.04 ^d^	15.11 ± 0.02 ^c^	14.03± 0.05 ^a^	71.71 ***	14.63 ± 0.02 ^a^	14.79 ± 0.04 ^a^	14.77 ± 0.03 ^a^	14.57 ± 0.01 ^a^	14.82 ± 0.02 ^a^	0.44 ns
pH	3.75 ± 0.10 ^c^	3.70 ± 0.08 ^bc^	3.69 ± 0.12 ^bc^	3.62 ± 0.12 ^ab^	3.55 ± 0.10 ^a^	8.335	3.82 ± 0.01 ^c^	3.67 ± 0.01 ^b^	3.53 ± 0.01 ^a^	3.60 ± 0.01 ^ab^	3.68 ± 0.01 ^b^	24.86 ***
Titratable acidity % lactic acid	0.17 ± 0.02 ^a^	0.25 ± 0.04 ^b^	0.31 ± 0.01 ^bc^	0.40 ± 0.1 ^d^	0.42 ± 0.07 ^c^	0.29 ***	0.22 ± 0.01 ^a^	0.34 ± 0.01 ^b^	0.30 ± 0.01 ^ab^	0.33 ± 0.01 ^b^	0.30 ± 0.02 ^ab^	0.08 * s 3.18 * s
Viscosity (Pa·s)	3.07 ± 0.04 ^b^	3.60 ± 0.03 ^c^	3.67 ± 0.05 ^c^	2.91 ± 0.04 ^b^	2.57 ± 0.05 ^a^	21.12 ***	3.08 ± 0.02 ^a^	3.19 ± 0.04 ^ab^	3.32 ± 0.04 ^b^	3.15 ± 0.07 ^ab^	3.08 ± 0.04 ^a^	1.17 ^ns^
Adhesives (J)	592.91 ± 43.3 ^c^	846.29 ± 194.73 ^a^	588.99 ± 113.23 ^c^	792.20 ± 107.13 ^ab^	683.52 ± 75.98 ^bc^	14.65 ***	691.94 ± 4.89 ^a^	720.14 ± 26.93 ^a^	646.54 ± 11.87 ^a^	700.58 ± 10.21 ^a^	744.73 ± 19.55 ^a^	0.82 ns
Hardness (g)	129.80 ± 10.91 ^a^	193.50 ± 50.34 ^b^	215.20 ± 49.36 ^b^	302.93 ± 49.59 ^c^	140.00 ± 25.39 ^a^	58.32 ***	208.13 ± 4.01 ^a^	192.10 ± 1.21 ^a^	181.00 ± 2.01 ^a^	201.86 ± 1.81 ^a^	198.33 ± 1.01 ^a^	0.30 ns
Cohesion	1.27 ± 0.17 ^bc^	1.28 ± 0.39 ^bc^	0.91 ± 0.33 ^a^	1.07 ± 0.45 ^ab^	1.48 ± 0.13 ^c^	6.93 ***	1.09 ± 0.18 ^a^	1.32 ± 0.06 ^a^	1.22 ± 0.08 ^a^	1.17 ± 0.08 ^a^	1.22 ± 0.04 ^a^	0.74 ns
Oat aroma	2.76 ± 0.20 ^a^	2.94 ± 0.02 ^a^	2.89 ± 0.15 ^a^	2.86 ± 0.09 ^a^	2.87 ± 0.13 ^a^	1.12 ns	2.84 ± 0.09 ^ab^	2.92 ± 0.07 ^ab^	2.78 ± 0.12 ^a^	2.76 ± 0.17 ^a^	3.02 ± 0.05 ^b^	4.81 **

ns—insignificant (*p* > 0.05), *—*p* < 0.05, **—*p* < 0.01, ***—*p* < 0.001, ^a–d^—different letters in the same line indicate significant differences between samples (*p* < 0.001); I M—yogurt without synbiotic microcapsules (control sample); I STH—yogurt containing synbiotic microcapsules with starch; I INU—yogurt containing synbiotic microcapsules with inulin; I G—yogurt containing microcapsules with glucose; I OLI—yogurt containing synbiotic microcapsules with oligofructose.

**Table 3:** Monitoring for color differences (ΔE) and opacity/brightness ΔC *.

	Storage Time (Days)	I.OLI	I.G	I.STH	I.INU
dE *	T_0_	0.79	0.71	0.65	1.15
	T_7_	0.22	0.36	0.66	0.56
	T_14_	0.56	1.30	1.09	0.32
	T_21_	0.55	1.02	1.05	0.48
	T_28_	0.43	0.98	1.04	0.52
dC *	T_0_	0.66	0.55	0.45	1.05
	T_7_	0.08	0.24	0.54	0.50
	T_14_	0.07	0.26	0.14	0.30
	T_21_	−0.02	0.01	0.14	0.38
	T_28_	−0.09	0.01	0.23	0.49

dE *—total color difference, ΔC *—opacity/brightness difference, I.INU—yogurt containing synbiotic microcapsules with inulin; I.OLI—yogurt containing synbiotic microcapsules with oligofructose; I.STH—yogurt containing synbiotic microcapsules with starch; I.G—yogurt containing microcapsules with glucose. dC *—opacity/brightness difference from the control sample.

## Data Availability

Not applicable.
